# Detyrosinated microtubules modulate mechanotransduction in heart and skeletal muscle

**DOI:** 10.1038/ncomms9526

**Published:** 2015-10-08

**Authors:** Jaclyn P. Kerr, Patrick Robison, Guoli Shi, Alexey I. Bogush, Aaron M. Kempema, Joseph K. Hexum, Natalia Becerra, Daniel A. Harki, Stuart S. Martin, Roberto Raiteri, Benjamin L. Prosser, Christopher W. Ward

**Affiliations:** 1 Department of Orthopaedics, University of Maryland School of Medicine, Baltimore, Maryland 21201, USA; 2 Department of Physiology, Pennsylvania Muscle Institute, Perelman School of Medicine, University of Pennsylvania, Philadelphia, Pennsylvania 19104, USA; 3 Department of Medicinal Chemistry, College of Pharmacy, University of Minnesota, Minneapolis, Minnesota 55455, USA; 4 Department of Informatics, Bioengineering, Robotics and System Engineering, University of Genova, Genova 16146, Italy; 5 Marlene and Stuart Greenebaum National Cancer Institute Cancer Center, University of Maryland School of Medicine, Baltimore, Maryland 21201, USA; 6 Center for Biomedical Engineering and Technology (BioMET), University of Maryland School of Medicine, Baltimore, Maryland 21201, USA

## Abstract

In striated muscle, X-ROS is the mechanotransduction pathway by which mechanical stress transduced by the microtubule network elicits reactive oxygen species. X-ROS tunes Ca^2+^ signalling in healthy muscle, but in diseases such as Duchenne muscular dystrophy (DMD), microtubule alterations drive elevated X-ROS, disrupting Ca^2+^ homeostasis and impairing function. Here we show that detyrosination, a post-translational modification of α-tubulin, influences X-ROS signalling, contraction speed and cytoskeletal mechanics. In the *mdx* mouse model of DMD, the pharmacological reduction of detyrosination *in vitro* ablates aberrant X-ROS and Ca^2+^ signalling, and *in vivo* it protects against hallmarks of DMD, including workload-induced arrhythmias and contraction-induced injury in skeletal muscle. We conclude that detyrosinated microtubules increase cytoskeletal stiffness and mechanotransduction in striated muscle and that targeting this post-translational modification may have broad therapeutic potential in muscular dystrophies.

## Abstract (web summary)

Microtubules are transducers of mechanical energy in muscle cells. Here, the authors show that mechanotransduction is regulated by post-translational detyrosination of microtubules in mouse heart and skeletal muscle, and that reducing detyrosination ameliorates symptoms in a model of Duchenne muscular dystrophy.

## Graphical abstract

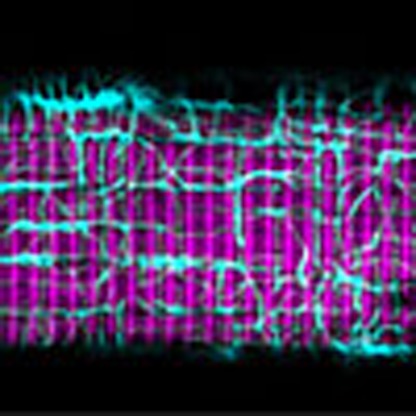

The cytoskeleton is critical for the cellular response to the mechanical environment, as it integrates and transduces mechanical energy to mechanosensitive proteins that generate biological signals. Known collectively as mechanotransduction, these pathways regulate diverse cellular physiology^1^ and dysregulation of these pathways is linked to disease^2^.

In striated muscle, microtubule (MT)-dependent mechanotransduction of reactive oxygen species (ROS) and calcium (Ca^2+^) signals has been characterized^3^,^4^,^5^,^6^,^7^. Termed ‘X-ROS' signalling, this mechanotransduction pathway links the mechanical stress of contraction or stretch to NADPH Oxidase 2 (NoX2) generated ROS signals that target Ca^2+^ channels^3^,^4^,^6^,^7^,^8^,^9^.

The MT contribution to X-ROS has gained considerable attention in the context of Duchenne muscular dystrophy (DMD), in which a dense and disorganized MT network accompanies severe striated muscle dysfunction^5^,^10^. In the *mdx* mouse model of DMD, we found that increased MT density^4^,^6^ and cytoskeletal stiffness^4^ correlated with excessive X-ROS that induced detrimental Ca^2+^ signals that are believed to underlie cardiac arrhythmia^6^,^9^ and contraction-induced damage in skeletal muscle^4^.

The MT network is a dynamic structure whose density is regulated by enzymes and binding proteins that affect the equilibrium between MT filament growth and disassembly^11^. In the *mdx* mouse, we demonstrated that an acute pharmacological strategy to promote MT disassembly (colchicine) reduced X-ROS and detrimental Ca^2+^ signalling *in vitro* and proffered therapeutic benefit *in vivo*, implicating increased MT density in the dysregulation of mechanotransduction in DMD. In light of transcriptional evidence from human DMD samples^4^ indicating an upregulation of the X-ROS pathway, as well as a link between MT density and X-ROS in additional dystrophy models^3^,^7^, we initially proposed that the increase in MT network density was a pathological factor in muscular dystrophy.

Assuming force transmission (F) through the cytoskeleton is a product of the stiffness (k) and deformation (x) of the cytoskeleton (F=kx), we reasoned that alterations in stiffness would regulate the magnitude of mechanotransduction. In this regard, we found detyrosinated α-tubulin^12^,^13^ a potentially attractive target for altering cytoskeletal stiffness. Detyrosination is the cleavage of a C-terminal tyrosine residue by an unidentified tubulin carboxypeptidase (TCP)^14^,^15^, a process readily reversed by tubulin tyrosine ligase (TTL)^16^,^17^. This modification stabilizes MT filaments by limiting disassembly^18^,^19^ and can increase the association of the MT network with other cytoskeletal elements^20^,^21^,^22^, which could increase cytoskeletal stiffness. Intriguingly, detyrosinated α-tubulin is abundant in disease and experimental conditions in which X-ROS is elevated^3^,^4^,^6^,^7^, yet its contribution to cytoskeletal stiffness and mechanotransduction remain undefined.

Here we report that MT detyrosination profoundly regulates mechanotransduction by modifying the mechanical properties of the cytoskeleton. By reducing detyrosination, we decreased cytoskeletal stiffness and increased the speed of muscle contraction and relaxation. Furthermore, these cytoskeletal changes dramatically reduced X-ROS signalling and activation of Ca^2+^ channels in both heart and skeletal muscle. These effects occurred without detectable changes in MT network density, indicating a specific role for detyrosination. Consistent with this reduction in X-ROS, we show that *in vivo* reduction of detyrosination in a murine model of DMD proffers significant protection from contraction-induced injury in skeletal muscle and workload-induced arrhythmia of the heart, both hallmarks of DMD.

We conclude that detyrosinated MT filaments affect the mechanosensitivity of striated muscle by altering the mechanical properties of the cytoskeleton. Given the limitations associated with colchicine treatment^23^, the identification of a specific subset of MTs that regulate cytoskeletal stiffness and mechanotransduction is a potentially viable therapeutic option. As this therapeutic strategy has already been established in cancer clinical trials^24^, targeting detyrosinated α-tubulin may have broad therapeutic potential for the treatment of muscular dystrophies.

## Results

### Striated muscles are enriched in detyrosinated MT filaments

**Figure 1:**
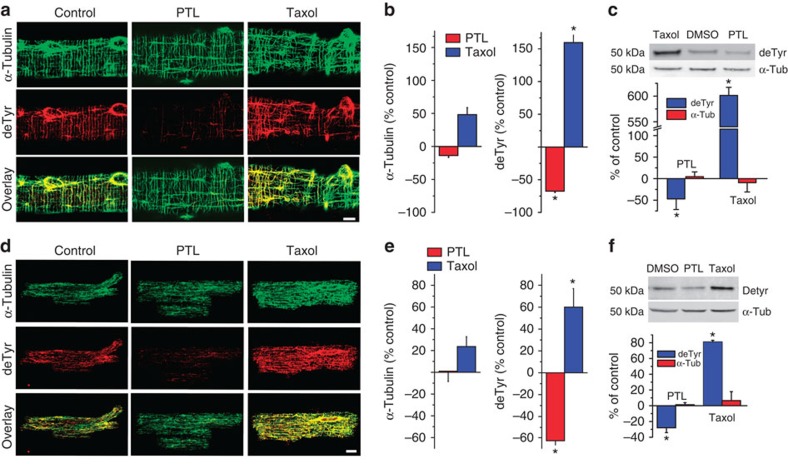
Detyrosinated α-tubulin is decreased following parthenolide treatment. (**a**) Skeletal FDB fibres isolated from WT rats were treated with PTL, taxol, or vehicle (control) before fixation, and immunostained for α-tubulin (green) and detyrosinated α-tubulin (red). (scale bar, 10 μm). (**b**) The density of the fraction of α-tubulin or detyrosinated α-tubulin was quantified (see Methods) following PTL (red) or taxol (blue) treatment and expressed as a per cent change from control (DMSO (dimethylsulphoxide)). PTL and taxol significantly decreased and increased detyrosinated tubulin, respectively. No significant differences in α-tubulin were identified with either treatment (*n*=7 fibres per group; *P<0.05). (**c**) Western blots of isolated FDB fibres showing that PTL significantly decreased, and taxol increased, the amount of detyrosinated tubulin, with neither treatment affecting the total α-tubulin content (*n*=5 mice; *P<0.05). Each treatment was normalized to DMSO control condition. (**d**,**e**) Cardiomyocytes isolated from WT rats treated with PTL, taxol, or vehicle control as above in A (scale bar, 10 μm) and quantified as in B. The detyrosinated MT fraction was significantly decreased with PTL and increased following taxol treatment, while the density of the total α-tubulin network was unchanged by PTL treatment and taxol treatment (*n*=8 cells per group; *P<0.05). (**f**) Western blots of cardiomyocytes revealed that PTL significantly decreased detyrosinated α-tubulin while taxol increased the amount of detyrosination. Neither treatment had an effect on total α-tubulin content (*n*=6 mice; *P<0.05). Blots quantitated as per cent change from controls. Differences between groups were determined by using a one-way analysis of variance followed by Dunnett's *post hoc* test. All values are mean±s.e.m.

Immunolabeling α-tubulin reveals a MT network favouring a fenestrated organization in skeletal *flexor digitorum brevis* (FDB) myofibres (Fig. 1a; control), and a longitudinal axis distribution in wild-type (WT) cardiomyocytes (Fig. 1d; control), consistent with previous reports^10^,^25^,^26^. Microtubule detyrosination is a post-translational modification (PTM) occurring post-polymerization, and estimates of total detyrosinated α-tubulin in various cell types range from 16-20% in epithelial cells, to approximately 60% in striated muscle, with a limited fraction (<10%) in the free tubulin pool^27^,^28^. Co-labelling cardiomyocytes and skeletal myofibres with an antibody against detyrosinated α-tubulin demonstrates that this PTM is abundant in WT cells and labels a subset of the total polymerized MT network (Fig. 1a,d).

Parthenolide (PTL) is a sesquiterpene lactone that inhibits the activity of the TCP^29^,^30^,^31^ and decreases the fraction of detyrosinated α-tubulin *in vitro*. A 2 hr treatment of isolated FDB myofibres or cardiomyocytes (Fig. 1a,d; PTL) with PTL results in an approximate 60% reduction in the amount of detyrosinated microtubules with no appreciable alteration in the density of the total network (Fig. 1b,e). Western blot verified this decrease in detyrosination, and confirmed that PTL did not alter the total α-tubulin content (Fig. 1c,f). As western blot measures both free and polymerized tubulin, and the majority of tubulin in striated muscle is in the free pool^10^,^32^, immunofluorescence is a more accurate indicator of the status of the polymerized network.

We also examined the effects of PTL on other common MT PTMs, namely, acetylation, polyglutamylation and the formation of Δ2 tubulin, all of which are associated with stabilized MTs with each being attributed to functional specificity in MT network regulation^33^,^34^. Although we detected polyglutamylation in FDB fibres and cardiomyocytes, the levels were unaltered by PTL treatment, as shown by western blot and immunofluorescence (Supplementary Figs 1,2). Acetylation was found in FDB fibres and levels were not altered by PTL, whereas little acetylation was detected in cardiomyocytes. We found Δ2-tubulin nearly undetectable in either cell type (Supplementary Figs 1,2). These results are consistent with a previous study of healthy adult myocytes^28^ and is suggestive of a unique role for detyrosination. Together, these results suggest that PTL specifically reduces the fraction of detyrosinated MT filaments, independent of filament density, tubulin content or other tubulin PTMs.

In contrast to PTL, taxol stabilizes MTs, increasing both network density and the proportion of detyrosinated MT filaments^27^,^35^. Treatment of FDB myofibres and cardiomyocytes *in vitro* with taxol (Fig. 1a,d; Taxol) significantly increases MT density and the degree of detyrosination by immunofluorescence (Fig. 1b,e). Western blots (Fig. 1c,f) further show that taxol increased the fraction of detyrosinated α-tubulin without altering total α-tubulin content. Taxol treatment also led to a modest increase in acetylation in skeletal myofibers, no detectable acetylation in cardiomyocytes, and little alteration to other PTMs in either cell type (Supplementary Figs 1,2) suggesting detyrosination as a potential contributor to taxol-dependent effects of MT function.

### Detyrosinated MTs regulate mechanical properties of muscle

**Figure 2:**
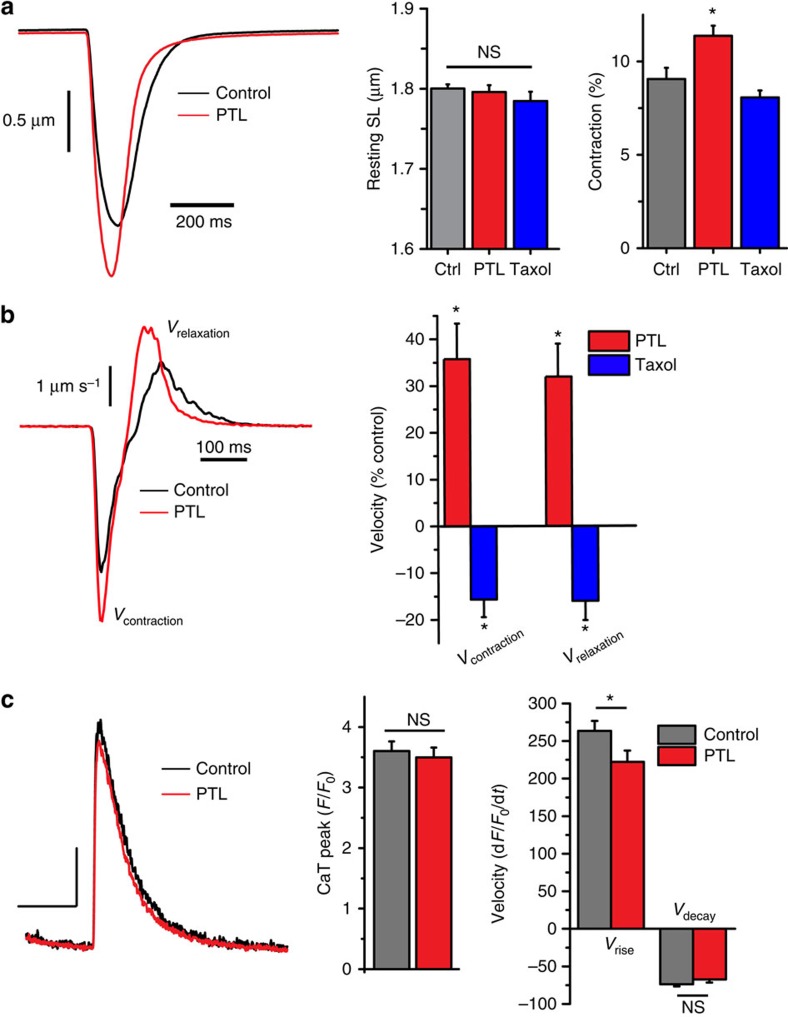
Contractile mechanics in cardiomyocytes are regulated by detyrosinated microtubules. (**a**) Sarcomere shortening was assayed in single cardiomyocytes under single action potential stimulation (1 Hz; field stimulation), contracting under no-load conditions (see Methods). SL measures of a control (black) and PTL (red) treated fiber are shown. Neither taxol nor PTL treatment significantly altered resting SL compared with the control (ctrl) condition. The per cent change (contraction %) in SL during contraction was significantly increased with PTL treatment but not by taxol. *P<0.05. (**b**) First derivative calculations of the SL shortening of a control (black) and PTL (red) treated cell expressed as a % from control. PTL significantly increased the velocity of SL shortening and relaxation while taxol decreased the velocity of each. *P<0.05 (*n*=36 control cells, 30 PTL cells, 30 taxol cells). (**c**) Ca^2+^ transients were assayed simultaneously with sarcomere mechanics following PTL treatment in single cardiomyocytes (*n*=5 cells per treatment). PTL (red) did not affect peak amplitude (measured by Fluo-3; see Methods) or the rate of decay of the global Ca^2+^ transient, and slightly slowed the rate of rise compared with the control condition (black/grey). *P<0.05 (*n*=38 control cells, 27 PTL cells). Statistical significance determined by one-way analysis of variance followed by Tukey *post hoc* test. All values are mean±s.e.m.

**Figure 3:**
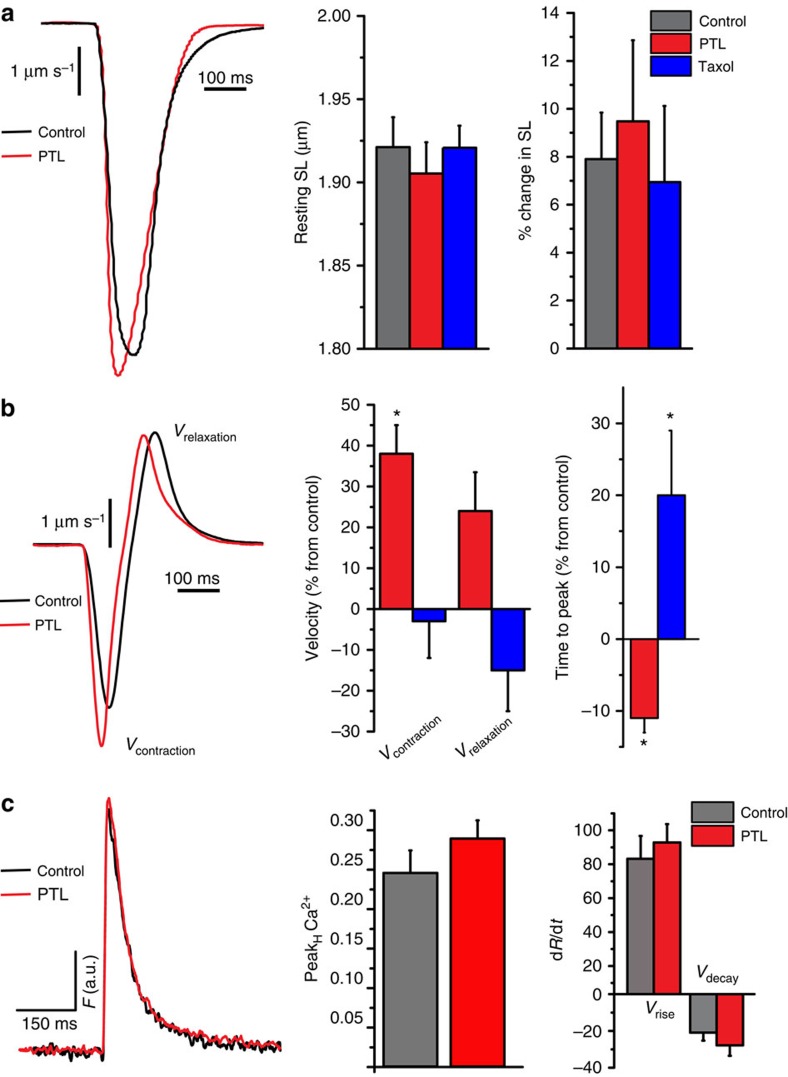
Mechanical properties of skeletal muscle are regulated by detyrosinated MT filaments. (**a**) Sarcomere shortening was assayed in single FDB fibres contracting with single action potential stimulation (1 Hz; field stimulation) under no-load conditions (see Methods). Representative raw SL measures of control (black) and PTL (red) treated fibres are shown. Neither taxol nor PTL treatment significantly altered resting SL or the per cent change (% change) in SL compared with the control condition. (**b**) PTL significantly increased the maximal velocity of contraction, although relaxation kinetics were unaltered. Taxol treatment did not significantly alter either contraction or relaxation velocity. However, PTL significantly decreased, while taxol significantly increased, the time to peak SL shortening. *P<0.05 (*n*=10 control, 18 PTL, 14 taxol). Statistical significance determined by using a Kruskal–Wallis with Dunn's *post hoc* test. (**c**) Following PTL treatment, Ca^2+^ transients (displayed as the resulting ratio, F(a.u.)) were assayed using the ratiometric dye, Indo1-PE during unloaded shortening of single FDB fibres (*n*=5 cells per treatment). PTL did not affect peak calcium or the kinetics of rise or decay. Statistical significance determined by Student's *t*-test. All values are mean±s.e.m.

MT detyrosination increases concurrently with the increased MT density that is suggested to suppress contractility in cardiac development^36^,^37^,^38^ and the failing heart^39^,^40^, and is dramatically increased in diseased skeletal muscle^4^,^5^,^7^. However, the distinction between MT network density and MT detyrosination *per se*, and how each contributes to cytoskeletal mechanics, remains unclear. We therefore examined sarcomere dynamics during paced contractions in cardiomyocytes and skeletal myofibres as a gross measure of the contribution of cytoskeletal properties to cellular mechanics. In cardiomyocytes, PTL treatment had no effect on the resting sarcomere length (Fig. 2a; SL) yet significantly increased the degree of sarcomere contraction (Fig. 2a; red) and the maximal velocity of contraction and relaxation (Fig. 2b; red), findings consistent with decreased cytoskeletal resistance. In contrast, taxol decreased the velocity of contraction and relaxation, consistent with increased cytoskeletal resistance (Fig. 2b; blue) as previously described^41^,^42^. In skeletal muscle fibres, neither resting SL nor the change in SL with contraction was significantly different following PTL or taxol treatment (Fig. 3a). The maximal rate of contraction during 1 Hz stimulation was increased with PTL, yet the relaxation rate was not significantly altered (Fig. 3a). However, the time to peak contraction was significantly increased with PTL and decreased with taxol (Fig. 3a), again consistent with the MT network contributing to cytoskeletal viscoelastic resistance. Measurements of calcium transients in PTL-treated cardiac and skeletal muscle cells indicate that changes in global calcium kinetics cannot explain the altered mechanics (Figs 2c and 3c), leaving a decrease in the viscoelastic properties of the cytoskeleton as the most plausible explanation for the increased contractility.

**Figure 4:**
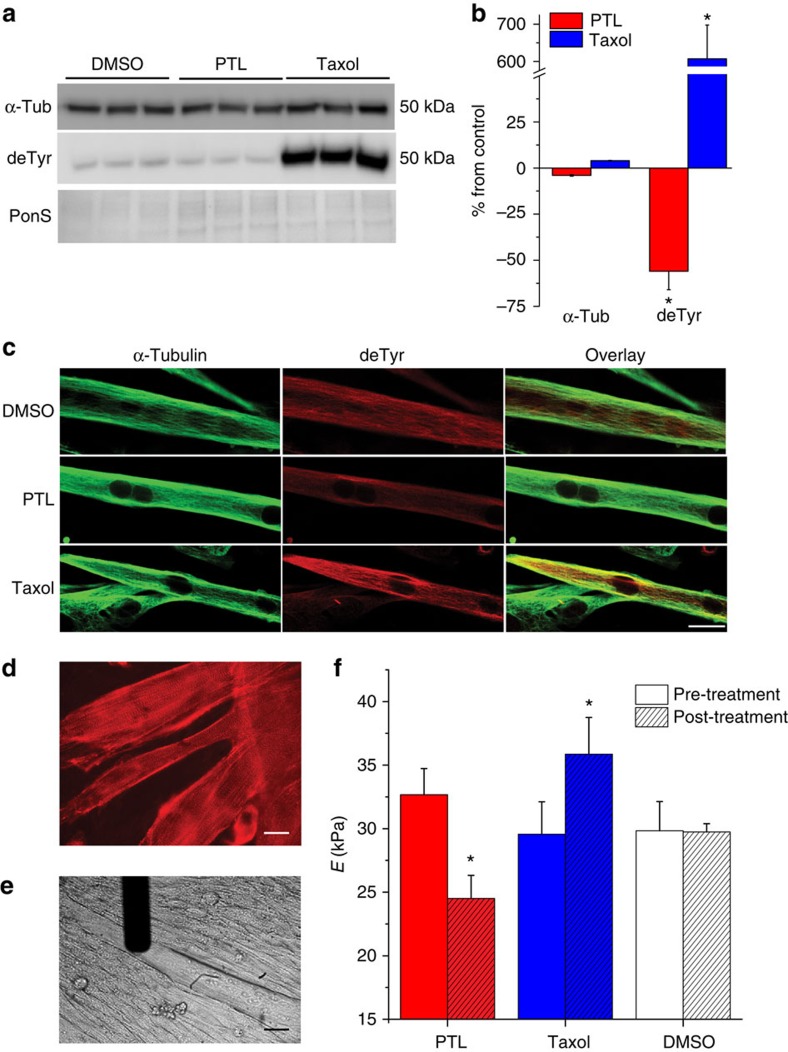
Detyrosinated tubulin affects cytoskeletal stiffness (**a**) Differentiated C2C12 cells were treated with either DMSO (dimethylsulphoxide), taxol (10 μM), or PTL (10 μM) before extraction for western blotting. Following PTL or taxol treatment, no change in α-tubulin was noted. In contrast, PTL significantly decreased and taxol significantly increased detyrosinated tubulin (normalized to control, as in (**b**)). *P<0.05 (*n*=6 per treatment) Statistical significance was determined by using a one-way analysis of variance (ANOVA) with a Tukey's *post hoc* test. (**c**) Differentiated C2C12 cells treated with DMSO, taxol, or PTL were fixed and immunostained for α-tubulin and detyrosinated tubulin. PTL decreased the amount of detyrosinated tubulin detected, while taxol increased detyrosinated MTs. Scale bar, 10 μm. (**d**) α-actinin staining confirming sarcomere development in differentiated C2C12 cells used for AFM measurements (Scale bar, 10 μm.) (**e**) Phase contrast showing AFM tip and C2C12 cells (Scale bar, 10 μm). (**f**) PTL treatment significantly decreased while taxol treatment significantly increased cytoskeletal stiffness. Unhashed bars indicate pre-treatment measures; hashed bars indicate measurements taken post-treatment. Statistical significance determined by repeated measures ANOVA with *post hoc* Tukey, *P<0.05 (*n*=12 cells per treatment) All values are mean±s.e.m.

We then directly tested the contribution of α-tubulin detyrosination to cytoskeletal stiffness by assaying near-membrane mechanical properties by atomic force microscopy (AFM)^4^. Differentiated skeletal C2C12 myotubes were used due to their robust adhesion to a substrate that permits paired pre- and post-treatment assessments of elastic moduli (Fig. 4d,e). We confirmed that the effects of both PTL and taxol on detyrosination in skeletal myotubes were similar to their respective effects on adult myofibres and myocytes, as shown via western blot (Fig. 4a,b) and immunofluorescence (Fig. 4c). In AFM studies, acute treatment with PTL significantly reduced, while taxol significantly increased, near-membrane transversal stiffness (Fig. 4f). Taken together, the differences in contractile mechanics and near-membrane stiffness support the conclusion that α-tubulin detyrosination can influence the overall mechanical properties of the cytoskeleton. The effects of PTL were independent of significant alterations in MT network density (see Figs 1b,e and 4a,b), suggesting that detyrosination can, by itself, modulate cytoskeletal mechanics.

### Detyrosinated MTs regulate X-ROS mechanotransduction

**Figure 5:**
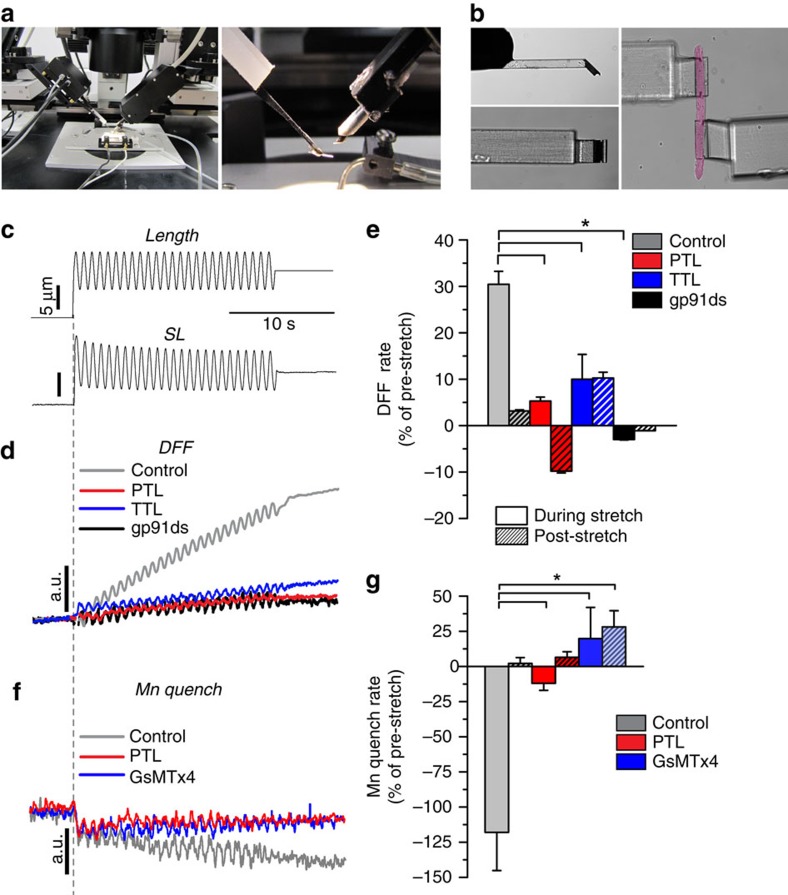
Mechano-activated ROS production and Ca^2+^ influx are regulated by detyrosinated tubulin. (**a**) Apparatus for the mechanical manipulation of single skeletal muscle fibres mounted over an inverted fluorescence microscope (left panel). Middle panel is close up view of myofibre holders mounted to the piezo length controller (left) and force transducer (right). (**b**) Microfabricated glass myofibre holders (kind gift of Ionoptix, Inc.; see Methods) have an etched channel (30 μm diameter; side view on top, bottom view below), that when coated with Myotak adhesive, are designed to receive and hold a single muscle fibre. The far right panel shows an isolated myofibre (pseudocolored pink) held by the microfabricated glass holders. (**c**) Single FDB myofibres were challenged with a protocol of dynamic sinusoidal stretch. Representative experimental records of piezo actuator length output (top) and SL(bottom) recorded in single control fibres. (**d**) Averaged fluorescence records of single FDB fibres loaded with the ROS indicator DFF at resting length (SL ∼1.83), during 20 s of dynamic sinusoidal stretch (8 μM length excursion at 1 Hz; from resting SL to ∼1.95 μm SL, approximately 8% SL excursion) and post stretch. (**e**) Dynamic stretch of control fibres (grey) elicited a significant increase in DFF fluorescence that returned to pre-stretch rates with the cessation of stretch (hashed bars). The Nox2 inhibitor peptide gp91ds-TAT ameliorated the mechano-activated ROS signal indicating X-ROS as the mechanism of ROS generation. Pre-treatment with PTL or overexpression of TTL significantly reduced the mechano-activated ROS signal. *P<0.05 (*n*=12 cells per treatment). (**f**) FDB fibres loaded with near-membrane Indo-1 were assayed for sarcolemmal Ca^2+^ permeability during mechanical stretch. The rate of decline in Indo1 fluorescence due to internalization of extracellular Mn and subsequent quenching of Indo1 fluorescence was taken as sarcolemmal Ca^2+^ permeability. (**g**) Compared with pre-stretch rates, dynamic sinusoidal stretch elicited a significant increase in Mn quench that was abrogated by PTL. The stretch-dependent Mn influx was abrogated by the mechanosensitive channel inhibitor GsMTx4. *P<0.05 (*n*=10 cells per treatment). Statistical significance determined by one-way analysis of variance with Tukey's *post hoc* test. All values are mean±s.e.m.

In our initial characterization of X-ROS, a small, brief stretch (∼10% SL, 5–10 s.) resulted in a burst of ROS in cardiomyocytes^6^, though this same protocol failed to evoke an X-ROS signal reliably in WT skeletal muscle fibres^4^. With refined methods and new fiber attachment technology (Fig. 5a,b), we now show that a repetitive sinusoidal length change (5 μm, 0.5 Hz; Fig. 5c) elicits a significant ROS signal in WT FDB myofibres (Fig. 5d,e; grey). The Nox2 inhibitory peptide gp91ds-TAT abolishes the generation of ROS during stretch (Fig. 5d,e; black), confirming that the ROS signal is indeed X-ROS.

Having established X-ROS in both skeletal muscle fibres and cardiomyocytes, we sought to determine the role of detyrosination in its generation. We found that skeletal muscle fibres treated with PTL had a significantly reduced X-ROS (Fig. 5d,e; red). To confirm this pharmacological approach, we overexpressed the highly-specific TTL (ref. 43), the enzyme responsible for the reversal of detyrosination by ligation of the C-terminal tyrosine residue on α-tubulin, by electroporation into FDB muscle. In fibres overexpressing TTL, we found a significant reduction in MT detyrosination (Supplementary Fig. 3) and stretch-dependent ROS generation *in vitro* (Fig. 5d,e; blue), firmly establishing that detyrosinated MTs promote the mechanotransduction of X-ROS.

In adult dystrophin-deficient skeletal muscle, excess X-ROS hyper-sensitized sarcolemmal Ca^2+^ influx channels to drive Ca^2+^ influx during stretch^4^. To test if X-ROS modulates Ca^2+^ influx in WT muscle, we used a modified manganese (Mn^2+^) quench assay and a near-membrane ratiometric calcium indicator (NM-Indo1) to determine sarcolemmal Ca^2+^ permeability during sinusoidal stretch. Following the replacement of extracellular Ca^2+^ with Mn^2+^ (Fig. 5f,g; grey), the stretch-dependent influx of Mn^2+^ through sarcolemmal cation channels quenched the NM-Indo1 signal. The mechanosensitive channel blocker GsMTx4 inhibited this Mn^2+^ quench (Fig. 5f,g blue), confirming the mechanosensitivity of this pathway. PTL treatment also significantly reduced stretch-dependent Mn^2+^ quench (Fig. 5f,g; red), supporting detyrosinated MTs as critical for the mechano-activation of X-ROS and subsequent calcium influx.

**Figure 6:**
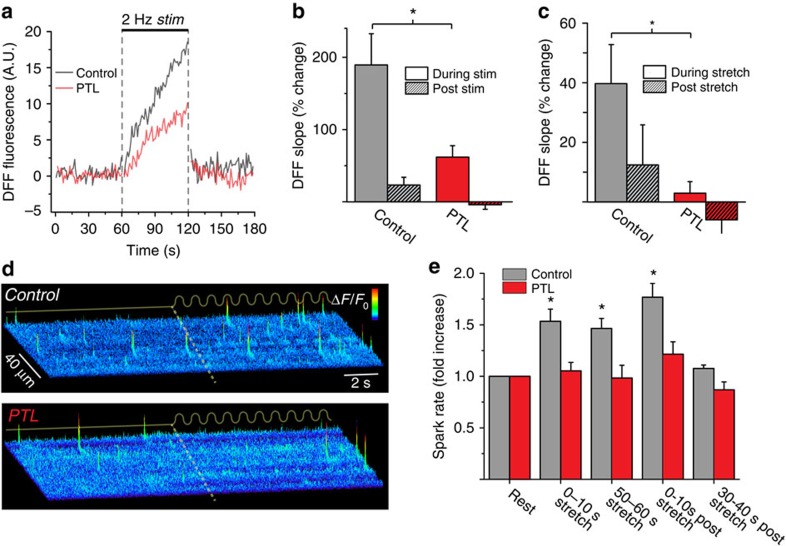
Mechanical stress-induced ROS production and Ca^2+^ sparks in heart. (**a**) Average traces (baseline subtracted, see Methods) of contraction-induced ROS production in isolated cardiomyocytes. (**b**) Parthenolide (PTL) reduces contraction-induced ROS production, quantified as per cent change in ROS production from baseline during (solid bars) and after (striped bars) 2 Hz field stimulation of isolated cardiomyocytes. *P<0.05 (*n*=49 control cells, 48 PTL cells). (**c**) PTL blocks stretch-dependent ROS production, quantified as per cent change in ROS production from baseline during (solid bars) and after (striped bars) 2 Hz 10% cyclic stretch of isolated cardiomyocytes. *P<0.05 (*n*=11 control cells, 12 PTL cells). (**d**) Representative fluorescence surface plots of calcium sparks in cyclically stretched myocytes with or without PTL treatment. PTL abrogated the stretch-dependent increase in calcium sparks seen in control cells. (**e**) Quantification of Ca^2+^ spark rate on first 10 s of initial cyclic stretch, after 1 min of sustained cyclical stretch, immediately following the release of stretch, and 30 s after the release of stretch, normalized to the pre-stretch rate *P<0.05 (*n*=22 control, 16 PTL cells). Statistical significance determined by repeated measures analysis of variance with *post hoc* Tukey. All values are mean±s.e.m.

In cardiomyocytes, X-ROS is generated with both stretch and electrically evoked contractions^6^,^9^. PTL treatment significantly reduced contraction-induced ROS production in cardiomyocytes, and eliminated ROS production with sinusoidal stretch (1 Hz, 10% of cell length) (Fig. 6a-c). X-ROS was previously demonstrated to increase the sensitivity of ryanodine receptor (RyR) calcium release channels, resulting in an increased frequency of Ca^2+^ ‘sparks'^6^, elementary calcium release events from the sarcoplasmic reticulum (Fig. 6d,e; control). Consistent with the inhibition of X-ROS, we found that PTL treatment dramatically inhibited Ca^2+^ sparks induced by sinusoidal stretch (Fig. 6e).

To control for non-specific effects of PTL treatment on the ROS-dependent regulation of calcium channels independent of MT and mechanotransduction, we applied H_2_O_2_ to cardiomyocytes and FDB fibres. H_2_O_2_ elicited a rapid increase in Ca^2+^ spark activity and Ca^2+^influx (Supplementary Fig. 4), independent of PTL. These data indicate that PTL directly interferes with the mechanotransduction through X-ROS, rather than altering Nox2 or ROS-dependent regulation of downstream targets.

### Reducing MT detyrosination protects DMD skeletal muscle

Growing evidence implicates a pathological role for altered MTs in cardiomyopathy^44^,^45^,^46^,^47^,^48^,^49^ and the muscular dystrophies^4^,^6^,^10^. Our group and others have demonstrated that acute pharmacologic disruption of the MT network with colchicine is effective at reducing disease-related signalling and functional pathologies in pre-clinical models^4^,^6^,^40^,^50^. However, the toxicity profile of this pharmacologic class^23^,^51^,^52^, underpinned by the interruption of essential MT network functions other than mechanotransduction (e.g., transport, signalling, metabolism), may limit the therapeutic potential of MT disruption.

Our previous work established that X-ROS drives detrimental Ca^2+^ signalling and pathology in heart and skeletal muscle from a mouse model of DMD (*mdx* mice), and that there is an increase in detyrosinated tubulin in both skeletal^4^ and cardiac^6^ *mdx* muscle. We confirm here, using our new sinusoidal stretch protocol, that *mdx* muscle fibres generate more X-ROS during stretch than WT controls (Supplementary Fig. 5). As in WT cells, acute treatment with PTL specifically reduces MT detyrosination and alleviates this excess X-ROS in *mdx* fibres (Supplementary Fig. 5). Based on this finding and the above *in vitro* evidence that reducing detyrosination inhibits X-ROS and its downstream consequences (Figs 5 and 6), we tested whether reducing detyrosination *in vivo* provided a significant benefit to the *mdx* mouse. We treated adult *mdx* mice (*n*=6) with LC-1 (or dimethylamino-parthenolide)^53^, a prodrug of PTL with enhanced solubility, or vehicle, once a day for three days. On the last day of treatment, mice from each group were evaluated for their resistance to either skeletal or cardiac stress-induced injury.

**Figure 7:**
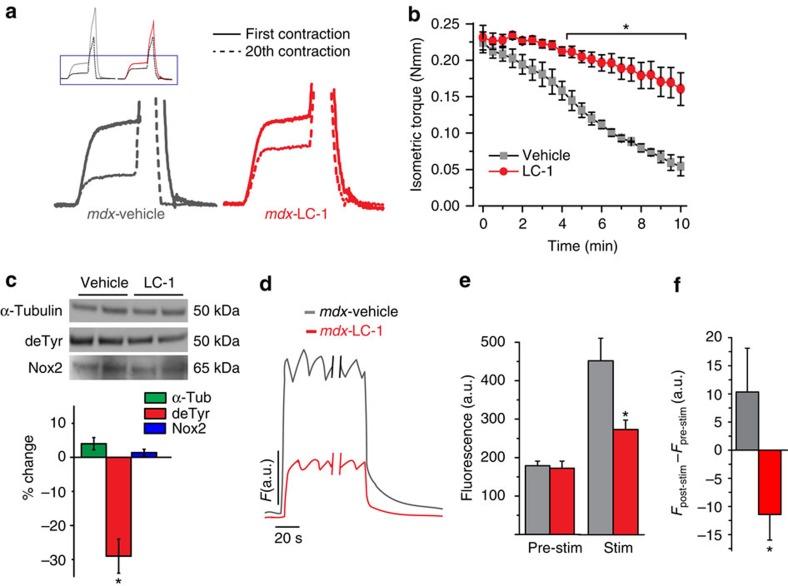
Targeting detyrosinated tubulin *in vivo* reduces contraction-induced force decline. *Mdx* mice (12 +/− 3.2 months) were treated with the PTL prodrug, LC-1 (100 mg kg^−1^) or vehicle control by oral gavage for 3 days. (**a**) On the fourth day, a protocol of 20 eccentric contractions revealed a decline in isometric force before stretch in both vehicle and LC-1 treated *mdx* mice. (**b**) Repetitive eccentric contractions of the gastrocnemius muscle were elicited *in vivo*. The characteristic decline in isometric force was significantly inhibited with LC-1 treatment. *P<0.05 (*n*=5). (**c**) Western blot of gastrocnemius muscles following LC-1 treatment show significant decrease in detyrosinated α-tubulin, but no change in Nox2 (gp91^phox^) or total α-tubulin content. Blots quantitated as per cent change from vehicle treatment. *P<0.05 (*n*=5) (**d**–**f**) FDB fibres from vehicle control or LC-1 treated mice were enzymatically isolated, loaded with calcium indicator dye (Fluo-4-AM). (**d**) Aggregate normalized Fluo4 traces during a sustained train of action potential pulses (20 Hz for 3 min). Ten sec before the cessation of stimulation, FDB myofibres from LC-1 treated *mdx* mice exhibited a significantly reduced peak cytosolic Ca^2+^. *P<0.05 (*n*=15 cells per treatment). (**e**) Quantitation of D. (**f**) Fibres from LC-1 treated *mdx* mice exhibited a significant increase in the decay of cytosolic Ca^2+^ following stimulation, as the post-stimulation fluorescence (*F*_post_) declined lower than pre-stimulation levels (*F*_o_) which yielded a negative *F*_post_-*F*_0_. In contrast, the *F*_post_ in vehicle-treated FDBs were significantly greater than pre-stimulation values 60 s after the end of the stimulation. *P<0.05 (*n*=15 cells per treatment). Statistical significance determined by Student's *t*-test. All values are mean±s.e.m.

Resistance to contraction-induced skeletal muscle injury was assessed *in vivo* by challenging anesthetized mice with 20 eccentric contractions of the hind limb plantar flexors^4^. The isometric torque of the initial contraction revealed no difference between the vehicle and LC-1 treated group, but subsequent contractions revealed a significant protection against contraction-induced force loss in animals treated with LC-1 (Fig. 7a,b). This level of protection was similar to our previous findings with pharmacologic ablation of the MT network or inhibition of Nox activity^4^. Western blot analysis of the contralateral uninjured muscle revealed that LC-1 treatment had no significant effect on Nox2 (gp91^phox^), α-tubulin content or levels of acetylated, polyglutamylated, or Δ2-tubulin, while decreasing the amount of detyrosinated tubulin to approximately WT levels (Fig. 7c, Supplementary Fig. 1)^4^.

We previously showed that ablation of the MT network can reduce detrimental mechano-activated [Ca^2+^]_i_ signalling in *mdx* fibres^4^, a hallmark of DMD. As a direct test of the effect of LC-1 treatment on contraction-induced Ca^2+^ signalling dysfunction, single *mdx* FDB myofibres were isolated from the foot of the uninjured leg of each treatment group, loaded with the fluorescent Ca^2+^ dye Fluo-4-acetoxymethyl (AM) and challenged with a 2 min, 5 Hz train of twitch contractions. Before stimulation, both groups exhibited a similar level of cytosolic fluorescence, however, during the 2 min test period, FDBs from LC-1 treated *mdx* animals maintained significantly lower cytosolic [Ca^2+^] compared with vehicle-treated *mdx* fibres (Fig. 7f,g). At the end of the pulse train, the return to resting [Ca^2+^] was significantly faster in LC-1 treated fibres, as [Ca^2+^]_i_ remained elevated above pre-stimulation values for upwards of 100 s following stimulation in untreated *mdx* fibres (Fig. 7h). Taken together, these results suggest that reducing detyrosination protects against contraction-induced Ca^2+^ signalling dysfunction and injury in *mdx* muscle.

### Reducing MT detyrosination protects DMD heart

**Figure 8:**
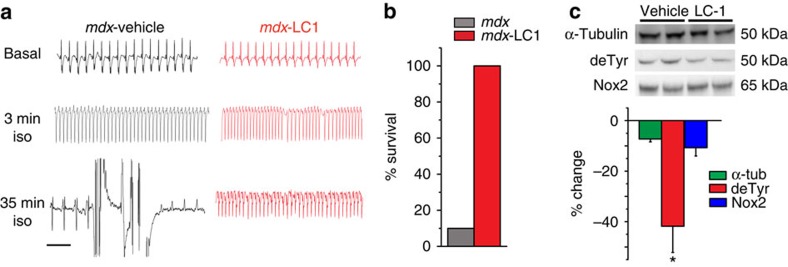
*In vivo* targeting of detyrosination in *mdx* heart rescues isoproterenol induced arrhythmia and lethality. (**a**) Representative ECG recordings from *mdx* mice given vehicle treatment or the parthenolide prodrug, LC-1, and exposed to an isoproterenol challenge to increase cardiac work. Off-scale readings in *mdx* trace represent agonal breathing shortly preceding death. (LC-1: *n*=6; vehicle: *n*=13) Scale bar, 1 s. (**b**) Per cent survival of mice following isoproterenol challenge. All 6 LC-1 treated mice survived (100% survival rate); 12 of 13 vehicle-treated mice were anesthetized due to fatal arrhythmia (7.6% survival rate). (**c**) Western blotting of isolated hearts following LC-1 or vehicle treatment shows a significant decrease in detyrosinated tubulin without significantly altering α-tubulin or Nox2 content. Blots quantitated as per cent change from vehicle-treated. Statistical significance determined by Student's *t*-test. *P<0.05 (*n*=3 per group). All values are mean±s.e.m.

We and others have previously shown that mechanical stress drives detrimental ROS and Ca^2+^ signalling in the *mdx* heart^6^,^54^ that can trigger arrhythmias^55^,^56^. We next assayed the contribution of the detyrosinated MT network to this pathology in the dystrophic heart. *Mdx* mice treated with either LC-1 or vehicle for three days were anesthetized, instrumented to record electrocardiogram (ECG) and challenged with the β-adrenergic agonist isoproterenol (0.5 mg kg^−1^) to increase cardiac work. Immediately following isoproterenol challenge, the resting heart rate increased two- to three-fold in both treatment groups. In vehicle-treated *mdx* mice (Fig. 8a), arrhythmias became apparent within 10 min and progressed in frequency until the mice succumbed to arrhythmia-related cardiac failure and death (mortality in 12 of 13 animals tested). In contrast, no mice in the LC-1 treatment group (*n*=6) succumbed to arrhythmia-related death (Fig. 8a). Western blot analysis of the vehicle versus LC-1 treated hearts revealed that LC-1 treatment had no significant effect on α-tubulin, acetylated tubulin, or Nox2 (gp91^phox^) content, but significantly decreased the amount of detyrosinated α-tubulin (Fig. 8c, Supplementary Fig. 1e). Consistent with LC-1 acting through X-ROS to exert its effect, protection from arrhythmia-associated death was also conferred by the *in vivo* inhibition of Nox2 with gp91ds-TAT (*n*=3/3 survival) or by pharmacologic disruption of the MT network with colchicine (*n*=5/5 survival).

**Figure 9:**
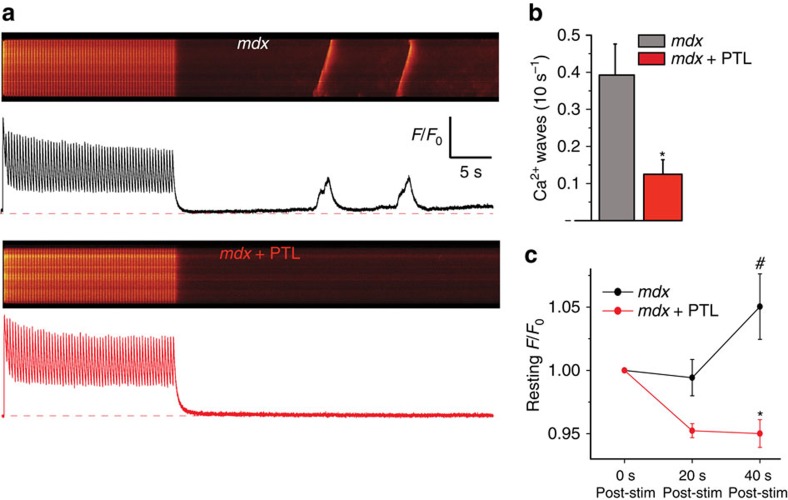
Parthenolide treatment ablates stress-induced Ca^2+^ waves and leak in *mdx* cardiomyocytes. (**a**) Representative traces of calcium transients and calcium waves during and after high-frequency (3 Hz) stimulation of *mdx* myocytes. (**b**) Parthenolide treatment significantly reduced the frequency of Ca^2+^ waves following high-frequency stimulation of *mdx* myocytes. (**c**) Parthenolide treatment prevented an elevation in resting calcium concentration following high-frequency stimulation of *mdx* myocytes *P< 0.01 versus *mdx* untreated, #*P*<0.05 versus *mdx* immediately post stim. (*n*=36 control cells, 36 PTL cells). Statistical significance determined by Student's *t*-test. All values are mean±s.e.m.

To directly test the role of MT detyrosination in generating arrhythmogenic calcium signals, we used an established high-frequency stimulation protocol to evaluate Ca^2+^-dependent arrhythmogenic potential in single *mdx* cardiomyocytes loaded with the Ca^2+^ indicator Fluo-3 (ref. 56). Following a 30 s burst of 3Hz electrical pacing, untreated *mdx* cardiomyocytes exhibited spontaneous, propagating Ca^2+^ waves linked to arrhythmia formation, as well as a significant increase in resting Fluo-3 fluorescence (Fig. 9a–c). These findings are consistent with RyR-mediated calcium ‘leak' that underscores arrhythmogenesis in *mdx* myocytes^6^,^55^,^56^. PTL treatment significantly reduced the frequency of Ca^2+^ waves and prevented the elevation in resting calcium concentration following high-frequency stimulation (Fig. 9a–c), suggesting that targeting MT detyrosination reduces detrimental calcium signalling and stress-dependent arrhythmogenesis in the DMD heart.

## Discussion

We report the novel discovery that detyrosination is a potent regulator of MT-dependent mechanotransduction in striated muscle. Using both pharmacologic and genetic strategies, we show that MT detyrosination correlates with the magnitude of X-ROS signalling in heart and skeletal muscle. As the level of detyrosination affects both the velocity of contraction and cytoskeletal stiffness, we conclude that detyrosination regulates mechanotransduction by altering the mechanical properties of the cytoskeleton.

Iribe *et al*^57^ first identified the role of MTs in the mechanotransduction of cardiac Ca^2+^ signalling. Subsequent studies identified X-ROS as the proximate activator of these Ca^2+^ signals in both cardiac^6^ and skeletal muscle^4^ by showing the blockage of X-ROS with pharmacologic ablation of the MT network. Here we report that a selective reduction in detyrosinated MTs yields a similar effect on mechanotransduction, while preserving the overall MT network. This was at first a surprising finding, particularly as there is no evidence that detyrosination contributes to the intrinsic stiffness of the individual MT filament *per se*, and historically, some have viewed this PTM (along with acetylation, polyglutamylation and Δ2-tubulin) as simply a marker of stable microtubules.

However, this view is being challenged, and our study adds to an emerging body of work demonstrating specific roles for detyrosination in the regulation of MT function^14^, including the organization of chromosomes in mitosis^31^ and the regulation of Na^+^/K^+^ ATPase activity^58^. We found detyrosination uniquely abundant in healthy muscle compared with other common PTMs (Fig. 1, Supplementary Figs 1,2)^28^, and PTL and LC-1 selectively reduced this PTM without detectably altering acetylation, polyglutamylation or Δ2-tubulin, allowing the attribution of functional consequences to detyrosination. On the other hand, taxol has complex effects^35^ as it increases the amount of polymerized MTs, and several tubulin modifying enzymes favour the polymerized form^14^. Here taxol increased MT network density, dramatically increased detyrosination, and modestly affected other PTMs, and thus results should be interpreted with this in mind. Separating the contribution of these individual PTMs to specific biological functions will be enabled by the continued identification of tubulin modifying enzymes and molecular tools to manipulate them, including the elusive TCP^14^.

How can reducing the fraction of detyrosinated MTs have such significant effects on mechanotransduction? While detyrosination may not alter intrinsic MT stiffness *in vitro*, it does increase MT stability by inhibiting disassembly via motor-proteins^18^. In addition, while MTs can resist compressive loads, the mechanical properties of the MTs alone are insufficient^59^, and likely require cross-linking interactions with other proteins and cytoskeletal elements to confer the requisite mechanical stiffness needed to resist cytoskeletal compression^60^. Recent studies linking functional deficits in heart failure and muscular dystrophy to the overexpression of Microtubule-Actin Cross-Linking Factor 1 (ref. 61) or the cytoskeletal cross-linker plectin^62^ offer support for this idea in striated muscle. Further evidence comes from non-muscle cells, where increased cytoskeletal stiffness is driven by interactions between detyrosinated MTs and intermediate filaments (IFs)^21^,^22^,^29^. In this context, it is notable that the expression of desmin, the major IF in striated muscle, is elevated in the *mdx* mouse^63^, and its deletion provides significant functional benefit^63^. While the authors did not attribute a rescue of dysregulated mechanotransduction to their results, our current data support this possibility, as increased MT:IF cross-linking offers a parsimonious explanation for the detyrosination-dependent elevations in X-ROS signalling and cytoskeletal stiffness. Finally, detyrosination also regulates the interaction of MTs with CAP-Gly proteins, which may have important consequences for both cytoskeletal organization and cell signalling^64^.

DMD provides an ideal model to test the contribution of detyrosinated MTs in mechanotransduction-dependent dysfunction, as an increase in MT network density and detyrosination correlates with pathology in DMD^4^,^5^,^6^,^10^. While previous studies have demonstrated that acute pharmacologic disruption of MT structure can be beneficial in rodent models of DMD and pathologic cardiac hypertrophy^4^,^32^, this strategy is limited by the potential for depressing other central MT functions^23^,^51^,^52^. Targeting detyrosination is an attractive strategy as it exerts significant effects while sparing the MT network. Here we report that acute *in vivo* pharmacologic reduction of detyrosination protects the DMD heart from workload-induced cardiac death and skeletal muscle from contraction-induced injury. Our *in vitro* studies confirm that reducing detyrosination inhibited the detrimental Ca^2+^ signals that underlie these DMD pathologies.

Although we limited our pre-clinical effort in this study to DMD, elevated MT density and detyrosination have been identified in multiple models of muscular dystrophy^4^,^5^,^7^,^8^ and pathologic cardiac hypertrophy^6^,^36^, suggesting that these MT alterations are conserved maladaptive responses in disease. Despite the growing evidence for a MT-targeted strategy to slow or halt disease progression in DMD and the failing heart, the move to clinical translation has been slowed by the unavailability of promising therapeutic targets and effective pharmacologic agents. PTL is a sesquiterpene lactone that has demonstrated promising utility as an anti-proliferative agent for treating human cancers^24^. Given that PTL (as feverfew extract) and LC-1 have advanced to Phase I human clinical trials^65^, compounds such as PTL represent novel lead candidates for MT-centric therapy in striated muscle disease. Beneficially, significant clinical data generated by cancer therapeutics^65^ will be available to guide the translation of SL therapy to striated muscle diseases.

In summary, we present evidence that MT detyrosination regulates mechanotransduction through ROS and Ca^2+^ signals. In diseased muscle sensitive to mechanical stress, inhibiting detyrosination blunts the stress-induced ROS and Ca^2+^ signalling dysfunction that drives pathogenic progression. We conclude that MT detyrosination regulates mechanotransduction in striated muscle and that targeting this PTM may have broad therapeutic potential in the muscular dystrophies.

## Methods

All reagents and drugs were purchased from Sigma-Aldrich unless otherwise noted. Gp91ds-TAT peptide was purchased from Anaspec. GsMTx4 was kindly provided by F. Sachs.

### Murine models

All animal care and experimental procedures were approved and performed in accordance with the ethical standards set by the Institutional Animal Care and Use Committees of both the University of Maryland and the University of Pennsylvania and by the National Institutes of Health (NIH). Mice (stock numbers 001801 and 000664) were acquired from Jackson Laboratories. Rats (Hsd:Sprague Dawley SD) were purchased from Harlan Labs. Animal cohorts within each genotype were randomly assigned to experimental groups.

### Preparation of flexor digitorum brevis (FDB) muscle fibres

FDB muscles from male control rat (WT), as well as male control mice (C57/Bl6; WT) and dystrophin-deficient (C57/10ScSn-*Dmd*^*mdx*^/J; *mdx*) mice at 9–11 months of age were isolated as follows: after euthanasia, FDB muscles were harvested bilaterally and placed in 4 mg ml^−1^ collagenase A. Following incubation at 37 °C for 2 h, the muscles were gently triturated to release individual fibres. Fibres were imaged or fixed within 24 h of isolation.

### Cardiomyocyte isolation

Adult C57/Bl6 or *mdx* mice and Sprague–Dawley rats were terminally anesthetized by injection of pentobarbital, followed by excision of the heart and enzymatic isolation of ventricular myocytes^66^. Hearts were suspended from a Langendorff perfusion column via cannulation of the aorta and perfused with cell isolation buffer containing in mM: NaCl 130, HEPES 25, Glucose 22, KCl 5.4, lactic acid 1, MgCl_2_ 0.5, NaH_2_PO_4_ 0.33, CaCl_2_ 0.3, to which insulin (100 micro-units per millimetre), collagenase II (0.66 mg ml^−1^ Worthington LS004176), trypsin (0.033 mg ml^−1^ Sigma T0303) and proteinase (0.033 mg ml^−1^ Sigma P8038) were added. Tissue was then minced and subjected to additional digestion with 0.7 mM CaCl_2_ and bovine serum albumin (2 mg ml^−1^) at 37 °C until myocytes were easily liberated by trituration with a Pasteur pipette. Myocytes were then removed from enzyme and then slowly reintroduced to physiological CaCl_2_ in cell isolation buffer without enzymes. Cardiomyocytes were stored in a normal Tyrode solution containing (in mM): NaCl 140, KCl 5, CaCl_2_ 1.8, MgCl_2_ 0.5, HEPES 5, Glucose 5 and NaH_2_PO_4_ 0.33. Experiments were performed at room temperature, 22 °C. Due to the relatively greater ease and reproducibility of highest quality cell preparations, rat myocytes were used for all experiments except when *mdx* myocytes and appropriate murine controls were needed.

### Immunofluorescence

Isolated FDB fibres were adhered to glass coverslips coated with Geltrex ECM (Life Technologies). Fibres were fixed at room temperature for 20 min using 4% paraformaldehyde in PBS, followed by permeabilization at room temperature for 15 min with 0.1% Triton X-100 in PBS (ref. 67). Non-specific staining was limited by adding SuperBlock PBS (Life Technologies) for 1 h before the addition of primary antibodies. Primary antibodies (α-tubulin clone DM1A, Sigma; rabbit detyrosinated tubulin, AbCam #48389; acetylated tubulin clone 6-11-B, Sigma; rabbit Δ2tubulin, Novus #NB100-57397; mouse polyglutamylation clone GT335, Adipogen) were diluted in SuperBlock PBS and added to the coverslips for an overnight incubation at 4 °C. Secondary antibodies (Goat anti-mouse Alexa 488, 647 and goat anti-rabbit Alexa 488, 568; Life Technologies; 1:100 dilution) were diluted in SuperBlock PBS and incubated at room temperature for 6 h. Coverslips were mounted on glass slides using ProLong Diamond (Life Technologies) and imaged using a Zeiss 510Live laser confocal microscope (× 63 1.4 numerical aperature plan-apo oil objective).

Isolated cardiac myocytes were fixed and permeablized in solution using 4% paraformaldehyde and 0.1% Triton X-100, both in PBS. Myocytes were then transferred to SuperBlock PBS and allowed to settle on coated coverslips for 1 h. The coverslips were then treated with antibodies, mounted and imaged as above.

Quantitation of α- and detyrosinated tubulin was performed with Volocity (Perkin Elmer). Total pixel density above threshold within a region of interest was calculated for each channel. The average density for each treatment condition and channel were calculated and normalized to the control untreated condition.

### Skeletal fibre mechanics and [Ca^2+^]_i_ transients

Isolated FDB fibres treated (2 h) with PTL (10 μM), taxol (10 μM), at room temperature, suspended in Hepes-buffered Ringer solution containing (in mM): 140 NaCl, 4 KCl, 1 MgSO_4_, 5 NaHCO_3_, 10 glucose and 10 HEPES (pH 7.3). Following treatment, fibres were placed in a custom rotating glass-bottomed perfusion chamber (Four-Hour Day Foundation) mounted over an inverted microscope (Olympus IX-70, × 40 objective). Contractions were elicited under no-load conditions with field stimulation (0.2-ms square pulse, 1 Hz elicited), while SL was monitored and recorded using a high-speed video SL system (HSVL 901B, Aurora Scientific). Across five successive contractions, velocity of shortening and relaxation as well as the time to peak shortening were calculated. Similarly, PTL or untreated FDBs equilibrated with the Ca^2+^ indicator Indo-1PE (5 μM, 0.1% Pluronic F127, 30 min) were electrically stimulated (1 Hz) and the peak, *V*_min_ and *V*_max_ of the Indo-1 ratio (405 nm/475 nm) were analysed (950 A, Aurora Scientific).

### Myocyte contraction and [Ca^2+^]_i_ transients

Experiments were performed in custom fabricated cell chambers (Four-hour Day Foundation, Towson, MD) mounted on a ZeissLSM 780 inverted confocal microscope with a × 40 Oil 1.4 NA objective. For measurements of contractility, SL was monitored with a high-speed video camera and Fourier transform analysis. The [Ca^2+^]_i_ transient was evaluated with Fluo-3 by 15 min incubation with 2 μM Fluo-3-AM ester and 0.01% Pluronic F127. Cells were allowed an additional 10 min for de-esterification and then scanned using a 488 nm argon ion laser in confocal line-scan mode at 1.92 ms per line. Cells were paced to steady state using 1 Hz field stimulation through platinum electrodes. After 20 s of 1 Hz stimulation, all cells showed steady state transients and contractions. At this point, five Ca^2+^ transients and five contractions were assayed. This protocol was repeated 2 × in each myocyte, and data was pooled for analysis.

### Myofibre attachment and stretch

All experiments were performed in a custom rotating glass-bottomed chamber (Four-Hour Day Foundation) equipped with bath perfusion and mounted on an inverted microscope (Olympus IX-70, 40 objective) fitted with a DG4 (Sutter Instruments), an IonOptix Myostretcher (IonOptix) system with a 403 A-CF force transducer (Aurora Scientific), and PMT Sub-System (IonOptix). Isolated FDB fibres were suspended in Hepes-buffered ringer as above, supplemented with PTL (10 μM) or left untreated (2 h). Individual fibres were attached between microfabricated glass fibre holders (IonOptix), coated with MyoTak biological adhesive (IonOptix), that were mounted to a force transducer (403A-CF, Aurora Scientific) and a Piezo actuator (PI). The force and positional outputs were recorded at 1kHz on a 600A data acquisition system from Aurora Scientific.

Stretch-induced ROS production was assayed in H_2_DFFDA (5-(and-6)-carboxy-2′,7′-dihydrofluorescein diacetate; 5 μM, 30 min; Life Technologies) loaded FDB fibres with a sinusoidal waveform (8% of resting SL) imposed for 20 s. Data were collected offline (500 Hz; 950A, Aurora Scientific) and the rate of ROS generation analysed using the Origin Pro 9.1 (Origin Lab). The Nox2-specific inhibitor gp91ds-TAT (2 μM) was used to verify the source of ROS.

Stretch-induced Ca^2+^ influx was assayed with a modified manganese quench (MnQ) assay. Briefly, cells were pre-loaded with Indo-1 Near-Membrane (NearMem, formerly FIP-18, 5 μM, Teflabs) and transferred and attached to the stretch apparatus described above. Perfusion with normal Ringer was switched to a Ringer where Ca^2+^ was substituted with manganese. Fibres were excited (340 nm), stretched with a sinusoidal waveform as above, and sarcolemmal permeability to Ca^2+^ estimated by the manganese influx and quench of the total emission fluorescence (*F*_TOT_=405+475 nm). The rate of fluorescence decrease was calculated and analysed as above. GsMTx4, a specific inhibitor of stretch-activated channels, was used to confirm the source of influx. For certain experiments, fibres were perfused with 200 μm H_2_O_2_ to elicit sarcolemmal calcium influx, independent of sinusoidal stretch (Supplementary Fig. 4).

### Cardiomyocyte attachment and stretch

Glass microrods were coated with a biological adhesive, MyoTak (IonOptix, Milton, MA). One glass microrod was connected to a force transducer (403A-CF, Aurora Scientific), and the other to a length controller (World Precision Instruments). Myocytes were attached at both ends by gently pressing down with the MyoTak-coated microrod and then lifting the cell from the chamber bottom. Axial stretch was applied by movement of the length controller in response to variable voltage output. SL was monitored with a high-speed video camera (Aurora Scientific). Average SL before stretch was 1.8 μm. Myocytes were subjected to three stretch-release trials, with 30–60 s rest allowed between trials. As each stretch produced a similar change in SL, DFF fluorescence, and Ca^2+^ spark rate, trials were pooled for analysis. WT cells did not commonly display Ca^2+^ waves, and cells with Ca^2+^ waves were discarded from analysis.

### Ca^2+^ spark measurements

Cells were loaded with Fluo-4 by 10 min incubation with 3 μM Fluo-4-AM ester and 0.01% Pluronic F127 and allowed an additional 10 min for de-esterification. Cells were scanned using a 488 nm argon ion laser in confocal line-scan mode at 1.92 ms per line. Automated analysis of line-scan images for Ca^2+^ spark location and properties was performed using SparkMaster (ImageJ^68^). For H_2_O_2_ studies, spark measurements were taken in the same cell before and after acute (<1 min) H_2_O_2_ (200 μM) exposure. Some cells were pretreated with PTL (2 h, 10 μM).

### DFF measurements in cardiomyocytes

Cells were loaded with the cell permeant ROS indicator DFF (a more photostable analogue to the commonly used DCF) by a 20 min exposure to a Tyrode's solution containing 2.5 μM H_2_DFFDA. The cells were imaged using confocal microscopy at 1 frame per second. Cells were imaged at very low laser intensity and with a sampling rest interval of 2 s to avoid artifactual signal amplification on continuous light exposure. The slope of the DFF signal is indicative of ROS production; therefore the slope before stretch has been subtracted from the entire trace so that the initial slope is zero and changes in slope can be easily visualized^4^,^6^. The slope is expressed normalized to the slope before stretch in that same cell as this comparison minimizes differences in DFF loading and resting signal between cells. As the slope was linear for the duration of cyclic stretch, the slope was calculated from the entire stretch duration for all cyclic stretch experiments.

### Atomic force microscopy

The transversal stiffness of differentiated C2C12 myotubes was quantitatively measured by means of nanoindentation measurements based on atomic force microscopy (AFM). Murine C2C12 myoblast cell line provides a well-established and reproducible model system for skeletal myogenesis^69^. When 80% cell confluence was reached, the medium containing 10% foetal calf serum was substituted with 2% foetal calf serum to induce myogenic differentiation, a process lasting a few days which can be recognized by a progressive elongation in cell shape and an assembling of structures involved in contraction. Differentiated C2C12 cells (American Type Culture Collection, ATCC) cultured on coverglass were treated for 2 h with PTL, Taxol or dimethylsulphoxide and then fixed and immunostained for MT as described above.

A commercial AFM (model 5500, Keysight Technologies) coupled with an inverted optical microscope was employed to record force-versus-distance curves, taken at a constant speed of 3 μm s^−1^ on differentiated C2C12 myotubes. At least three 500 × 500 nm^2^ areas, selected over the cell body to avoid nuclei regions, were sampled with a regular grid of 16 × 16 force curves each, for a total number of force curves ≥768 for each considered cell. The same commercial silicon microcantilever with a conical tip (model CSG11, NT-MDT, nominal spring constant *k*=0.03 N m^−1^) was used for all measurements. The region after contact of each force curve was transformed into a load-versus-indentation curve and the corresponding contact stiffness could be calculated at a certain maximum indentation. To maximize the sensitivity of the measurement towards the microtubule network structure and organization the reported values were calculated at a maximum indentation of 100 nm. Acute treatments were performed by incubating the already differentiated C2C12 culture for 2  in the medium containing either parthenolide or taxol at a final concentration of 10 μM. Before starting the AFM measurements the solution was replaced with fresh medium. Following AFM indentation measurement, cultures were labelled by using a primary antibody against α-actinin (clone EA-53, Sigma; goat anti-mouse Alexa 549 secondary) and fluorescent phalloidin (Alexa 488) to confirm the periodic sarcomeric organization of striated muscle fibres.

### Formulation of LC-1

The parthenolide prodrug LC-1 (ref. 53) was formulated for oral dosing in 2-hydroxypropyl-β-cyclodextrin (HP-β-CD)^70^. In brief, 160 mg of LC-1 were dissolved in 3.75 ml of an aqueous 0.3 M HP-β-CD solution and mixed well. The LC-1 and vehicle only solutions were stored at 4 °C before administration.

### *In vivo* injury

*Mdx* mice (9–11 months) were treated daily via gavage with a bioavailable parthenolide analogue (LC-1) or vehicle (HP-β-CD) for 3 days (100 mg kg^−1^). On the following day, mice were anesthetized and eccentric injury of the gastrocnemius was performed using a 305B muscle lever system (Aurora Scientific Inc.)^4^. The knee was stabilized and the foot firmly affixed to a footplate on the torque sensor. Contraction was elicited by percutaneous electrical stimulation of the sciatic nerve. Optimal isometric tetanic torque was determined with increasing the current, with a minimum of 30 s between each stimulation to prevent fatigue. Eccentric contractions were created by translating the footplate 30° backward at a velocity of 40 mm s^−1^ after the first 200 ms of the isometric contraction. Eccentric contractions at maximal isometric torque (0.2-ms pulse train at 100 Hz) were assayed 20 times with 1 min pauses between each, for the resistance to muscle damage. The decrease in the peak isometric force before the eccentric phase was used as a measure of damage. Following injury, the gastrocnemius was excised and flash frozen for western blotting.

### *In vivo* manipulation of cardiac workload

Mice (9–11 months) were treated with either LC-1 or vehicle (HP-β-CD) for 3 days (100 mg kg^−1^). On the subsequent day, mice were lightly anesthetized and instrumented for non-invasive ECG (Biopaq; three lead). ECG was monitored and digitized (1KHz) for 5 min before a challenge with isoproterenol (ISO; 0.5 mg kg^−1^) and then for 45 min or until the heart rate was significantly depressed, at which time the mouse was euthanized under anaesthesia. Hearts were excised and flash frozen for western blotting.

### Calcium stress assay

FDB muscle fibres from LC-1 and vehicle-treated mice were isolated as described above, placed into Hepes-buffered Ringer's solution and plated into ECM-coated wells of a clear-bottom 96-well plate. Cells were loaded with Fluo-4 (5 μM) for 30 min and rinsed with Ringer's solution. The cells of individual wells were electrically stimulated using a custom perfusion and stimulation apparatus (Four-Hour Day Foundation) for 5 min at 20 Hz, during which time Ca^2+^ was recorded.

### Western blotting

For western blotting, 20 mg of clarified muscle or C2C12 extract was subjected to SDS-polyacrylamide gel electrophoresis, transferred on nitrocellulose membranes, and washed/blocked in 5% milk solution in PBS for 1 h. The membrane was probed overnight with primary antibody at room temperature. The primary antibodies were: anti-α-tubulin (Sigma DM1A, 1:1,000), anti-detyrosinated tubulin (AbCam #ab48389, 1:1,000), anti-acetylated tubulin (Sigma 6-11-B, 1:200), anti-Δ2-tubulin (Novus ##NB100-57397, 1:1,000), anti-polyglutamylation (Adipogene GT335, 1:1,000) and anti-gp91^phox^ (BD Transduction Labs #611415, 1:1,000). Membranes were washed two times for 10 min in 5% milk solution at room temperature and incubated with appropriate secondary antibody (1:10,000) for 1 h at room temperature, and the membranes were washed in 0.5% Tween solution in PBS two times for 10 min. Membranes were then exposed to enhanced chemiluminescence with SuperSignalWest Pico Chemiluminescent Substrate to develop immunoblots. Blots were imaged and quantified with an imaging system (Syngene, G:Box; GeneTools software), normalized to total protein (via PonceauS) or GAPDH (Fig. 1, cardiomyocytes) and analysed using ImageJ. Images were cropped for presentation. Uncropped scans of western blots are shown in Supplementary Fig. 6.

### Statistical analysis

The sample number for each experiment was determined by a power analysis (http://www.power-analysis.com/) assuming a Cohen's maximum variability of the population mean (determined by variance in published experiments) and a type 1 error of 0.5%. For *in vivo* studies, animals were randomly assigned to treatment groups and blinded for experimentation and analysis. All other experiments were performed and analysed unblinded. Normality of data sets was ensured for each statistical calculation. Significance was set *a priori* at *P*<0.05.

## Additional information

**How to cite this article**: Kerr, J. P. *et al*. Detyrosinated microtubules modulate mechanotransduction in heart and skeletal muscle. *Nat. Commun*. 6:8526 doi: 10.1038/ncomms9526 (2015).

## Supplementary Material

Supplementary Information Supplementary Figures 1-6
