# Supplementary material for: Detyrosinated microtubules modulate mechanotransduction in heart and skeletal muscle
Source: Nat Commun. 2015 Oct 8;6:8526. doi: 10.1038/ncomms9526 (PMC4633818; doi:10.1038/ncomms9526)
Supplement: Supplementary Information — Supplementary Figures 1-6 [file ncomms9526-s1.pdf]

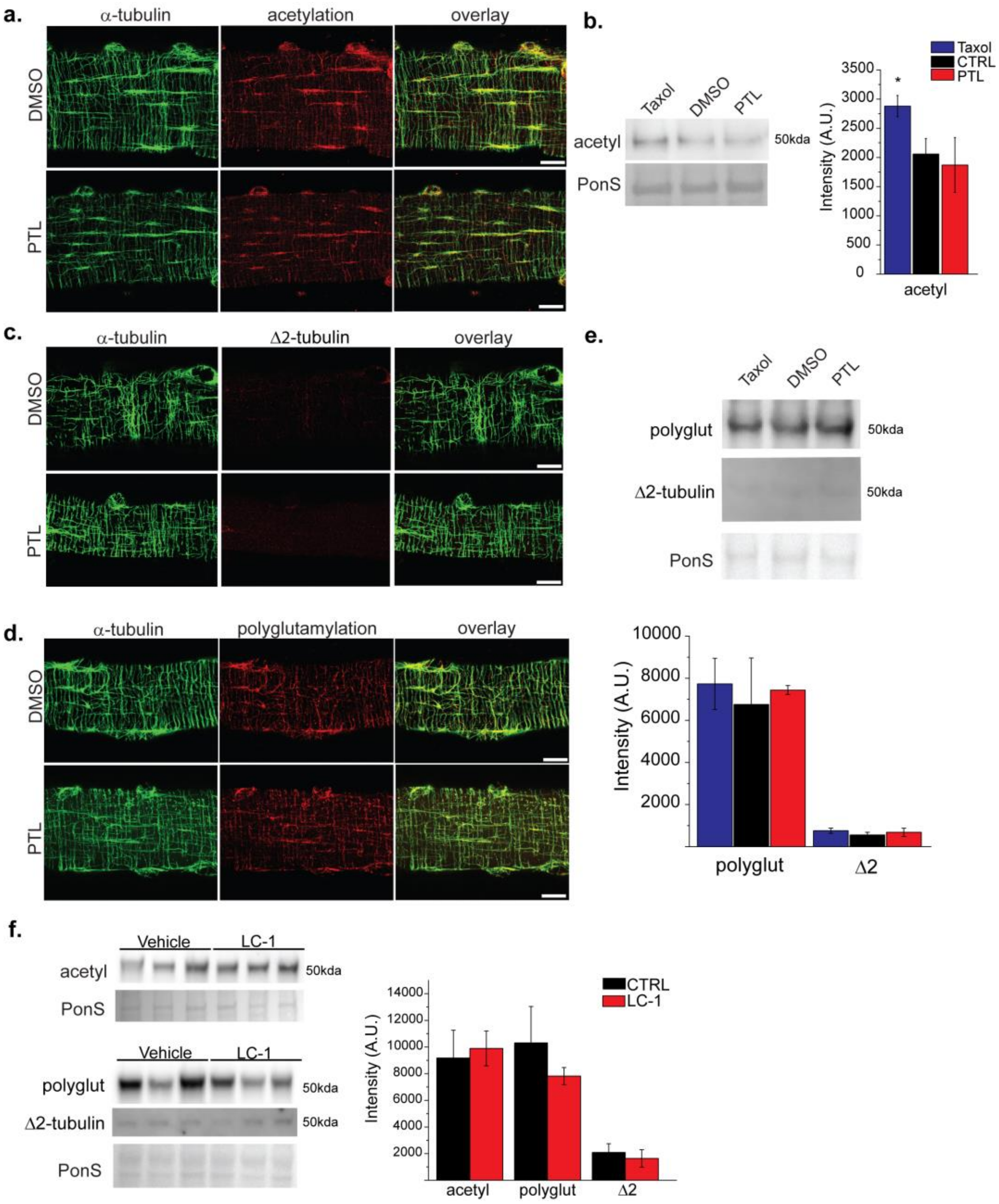

Supplementary Figure 1: Other tubulin PTMs are not affected by PTL treatment in skeletal muscle. **a.** Isolated WT skeletal myofibers (FDB) immunostained for α-tubulin and acetylated tubulin revealed the presence of acetylated MTs (DMSO, top). Microscope settings were adjusted for the control group, and kept constant between treatments to allow comparison. Quantitation of α- and acetylated tubulin was performed with Volocity (Perkin Elmer). Total pixel density above threshold within a region of interest was calculated for each channel. The average density for each treatment condition and channel were calculated and normalized to the control untreated condition. Acetylation was not visibly altered by PTL treatment. (n = 5 cells per treatment) Scale bar = 10µm. **b.** Western blots of WT FDB fibers treated with Taxol, PTL, or control (DMSO) indicate that, while taxol treatment leads to a moderate increase in acetylation (40%

increase over control), PTL has no effect, confirming immunofluorescence results. *$P<0.05$ for taxol v. control (n = 3 per group). **c.** Immunostaining of WT FDB fibers revealed no detectable Δ2-tubulin. (n = 3 per treatment) Scale bar = 10µm. **d.** Immunostaining of WT FDB fibers revealed polyglutamylated MTs (DMSO, top) that were not visibly altered by PTL. Microscope settings set for the control group, and were kept constant between treatments to allow comparison. (n = 5 per treatment) Scale bar = 10µm. **e.** Western blots of WT FDB fibers treated with Taxol, PTL, or control (DMSO) confirmed the expression of polyglutamylated tubulin that is unaltered by treatment and verify the limited level of Δ2-tubulin. $P=0.68$, One-Way ANOVA (n= 3 animals per group). **f.** Western blots of muscle extracts derived from *mdx* mice treated with either vehicle or LC-1 for 3 days. While detyrosination was significantly decreased (Fig. 7c), no significant changes in acetylated, Δ2, or polyglutamylated tubulin were detected. $P=0.48$ for polyglutamylation, $P=0.45$ for Δ2-tubulin, and $P=0.74$ for acetylation, via Student's t-test (n= 3 animals per group). Data presented as mean±S.E.M.

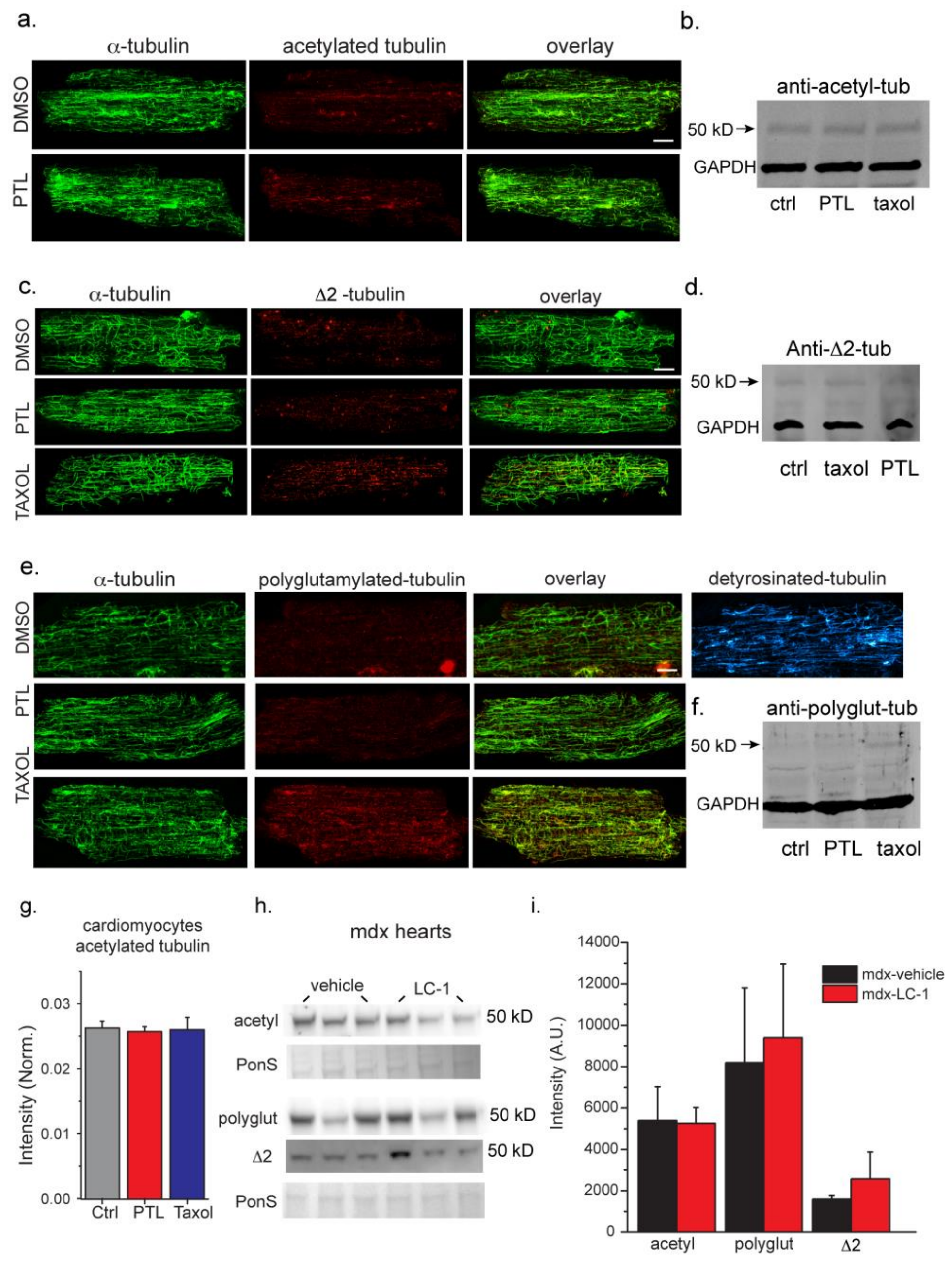

Supplementary Figure 2: Other tubulin PTMs are not affected by parthenolide (PTL) treatment in cardiac muscle. **a.** Isolated cardiomyocytes immunostained for α-tubulin and acetylated tubulin demonstrate a low level of acetylated MTs (DMSO, top) that are not visibly altered by PTL treatment. Microscope settings were adjusted for the control group, and kept constant between treatments to allow comparison. Scale bar = 10µm. **b.** and **g.** Western blots of cardiomyocytes show that acetylation is not altered by taxol or PTL treatment **c.** Immunostaining of cardiomyocytes revealed a low to undetectable level of Δ2-tubulin. Scale bar = 10µm. **d.** Western blot verified the low to undetectable level of Δ2-tubulin in these cardiomyocytes. **e.** Immunostaining of cardiomyocytes also revealed a low to undetectable level of polyglutamylated α-

tubulin. This relative difference in the abundance in polyglutamylation vs. detyrosination is evident in a cardiomyocyte co-stained for detyrosinated α-tubulin (pseudocolor blue). Scale bar = 10μm. **f.** The low abundance of polyglutamylation was verified by Western blot, where a band could be detected with taxol treatment, but only when contrast was increased to saturate GAPDH signal. Note that 3x more protein was loaded for panels b, d, and f compared to tubulin blots in Fig. 1f, as this was required to detect acetylation, Δ2, or polyglutamylation signal. **h.** Western blots of muscle extracts derived from *mdx* mice treated with either vehicle or LC-1 for 3 days. While detyrosination was significantly decreased (see Fig. 8b), no significant changes in acetylated, Δ2, or polyglutamylated tubulin were detected. P=0.26 for Δ2-tubulin, P=0.70 for polyglutamylation, and P=0.93 for acetylation via Student's t-test (n=3 animals per group). Data presented as mean±S.E.M.

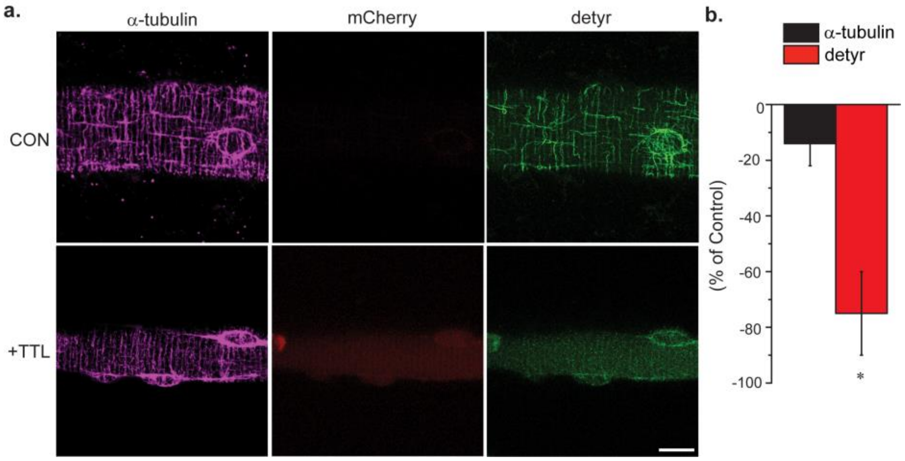

Supplementary Figure 3: Tubulin Tyrosine Ligase (TTL) overexpression decreases detyrosination in skeletal myofibers. *Flexor digitorum brevis* (FDB) muscles were electroporated with TTL-IRES-mCherry cDNA to overexpress TTL. **A.** Myofibers were then isolated, fixed, and immunostained for both α- and detyrosinated tubulin. Scale bar = 10µm. **B.** In myofibers expressing TTL (identified by mCherry expression), detyrosination (measured by pixel intensity within an ROI using Volocity; see Methods) was significantly decreased (75% reduction compared to control fibers not expressing TTL; $^*P<0.05$, $n=5$) while α-tubulin expression was unaltered (14% reduction compared to control fibers not expressing TTL; $P=0.177$; $n=5$). Statistical significance was determined by Student's t-test. Data presented as mean±S.E.M.

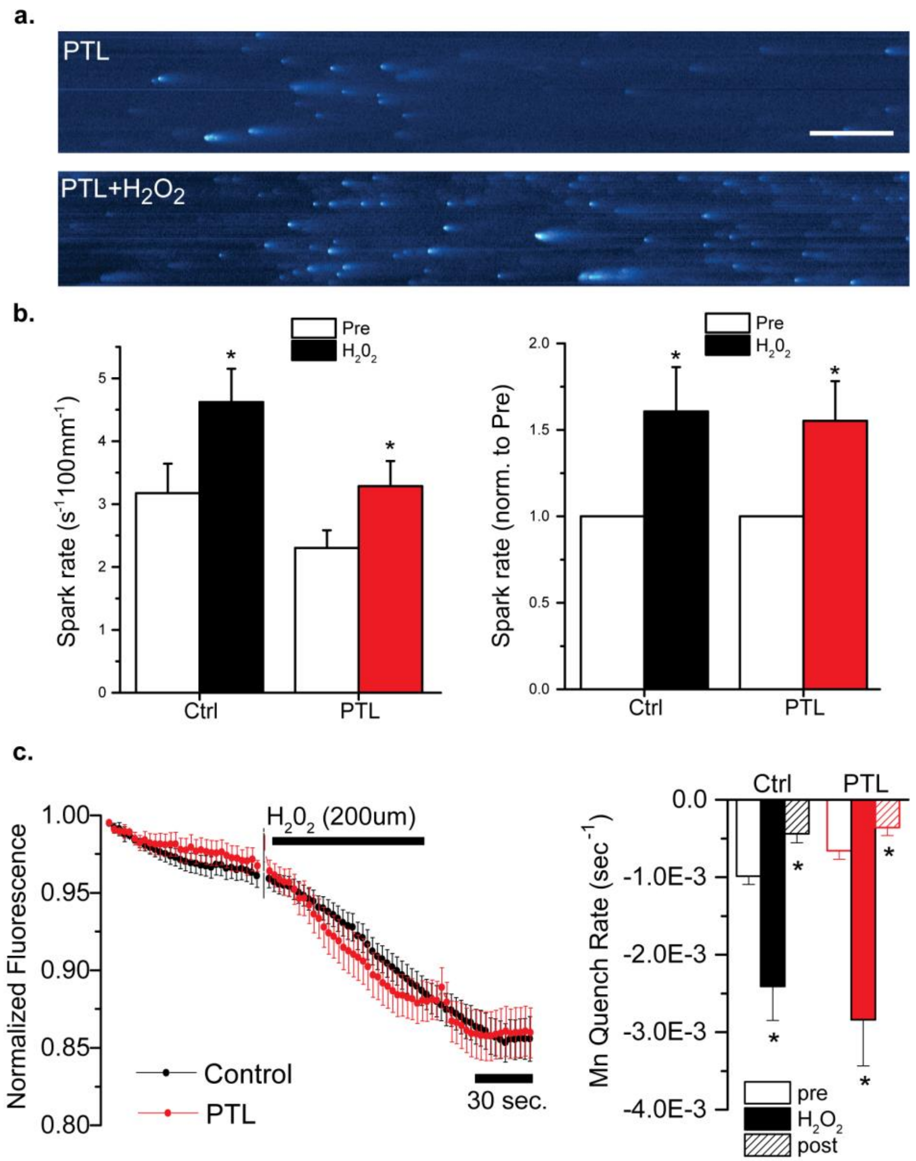

Supplementary Figure 4: PTL treatment does not alter the oxidation potential of downstream Ca^{2+} effectors. **A.** Representative x vs. t line scan recordings of calcium sparks in a myocyte pretreated with PTL, before and 30s after the addition of 200 μm H_2O_2. Scale bar = 1 s. **B.** Quantification of calcium spark rate in ctrl (n = 7) and PTL (n = 11) myocytes before and after H_2O_2 – left panel shows absolute spark rate, right panel is normalized to the spark rate within that cell prior to H_2O_2. *P<0.05. **C.** FDB fibers loaded with near-membrane Indo-1 were assayed for sarcolemmal Ca^{2+} permeability as in Figure 5, but with 200μm H_2O_2. Quantification shows that the ROS induced sarcolemmal Ca^{2+} permeability is unchanged in fibers pre-treated with PTL. *P<0.05 (n = 32 ctrl, 38 PTL fibers). Statistical significance determined by one-way ANOVA with post-hoc Tukey. Data presented as mean±S.E.M.

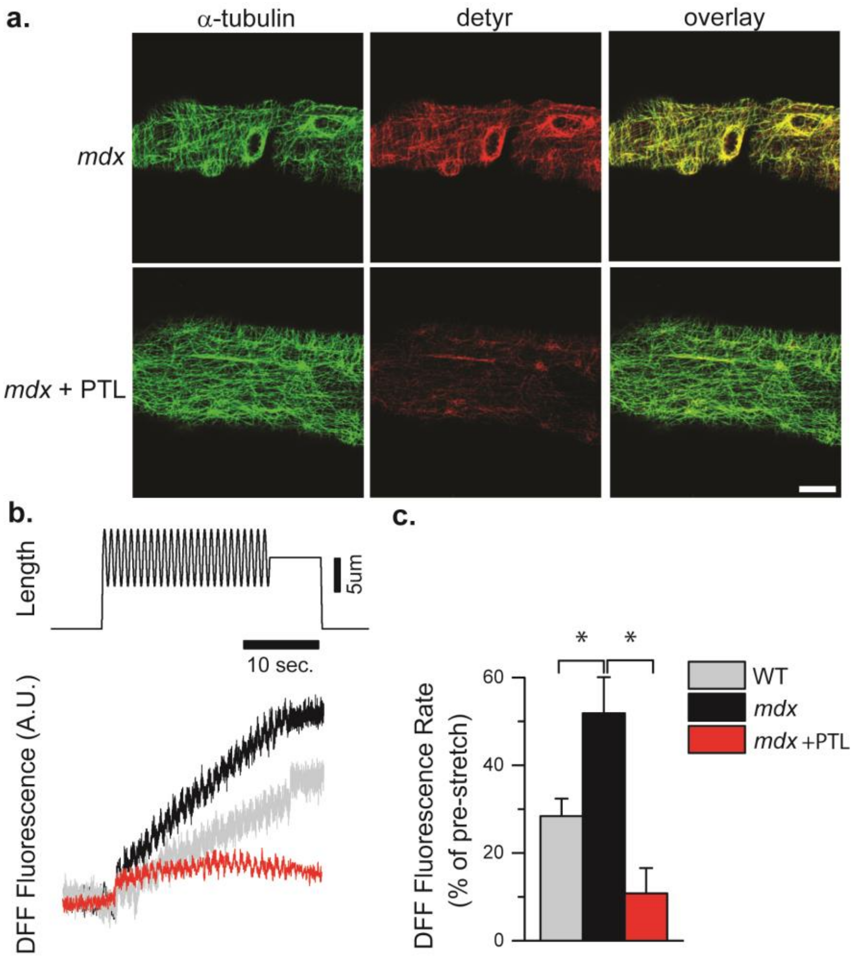

Supplementary Figure 5: <u>PTL reduces X-ROS in *mdx* muscle fibers.</u> **A.** Isolated myofibers from *mdx* FDB muscles were treated with PTL (10µM; 2hr) or untreated. Myofibers were then fixed and immunostained for α-tubulin and detyrosinated tubulin. PTL treatment reduced detyrosinated tubulin, but did not affect MT disorder. (n=5 cells per treatment) Scale bar = 10µm. **B.** Single FDB myofibers from *mdx* were challenged with a protocol of dynamic sinusoidal stretch. Representative experimental records of piezo actuator length output (top) and averaged fluorescence records of single *mdx* FDB fibers loaded with the ROS indicator DFF at resting length (SL ~1.83), during 20 sec of dynamic sinusoidal stretch (8 µM length excursion at 1Hz; from resting SL to ~1.95 µm SL, approximately 8% SL excursion) and post stretch (bottom). WT trace is aggregate as seen in Fig. 5. **C.** Dynamic stretch of WT fibers (gray) elicited a significant increase in DFF fluorescence. Stretch of *mdx* myofibers (black) elicited an increased DFF fluorescence compared to controls. Pre-treatment of *mdx* myofibers with parthenolide (PTL; red) significantly reduced the mechano-activated ROS signal. *P<0.05 as determined via One-Way ANOVA with Tukey's *post hoc* test (n = 5 cells per treatment). Data presented as mean±S.E.M.

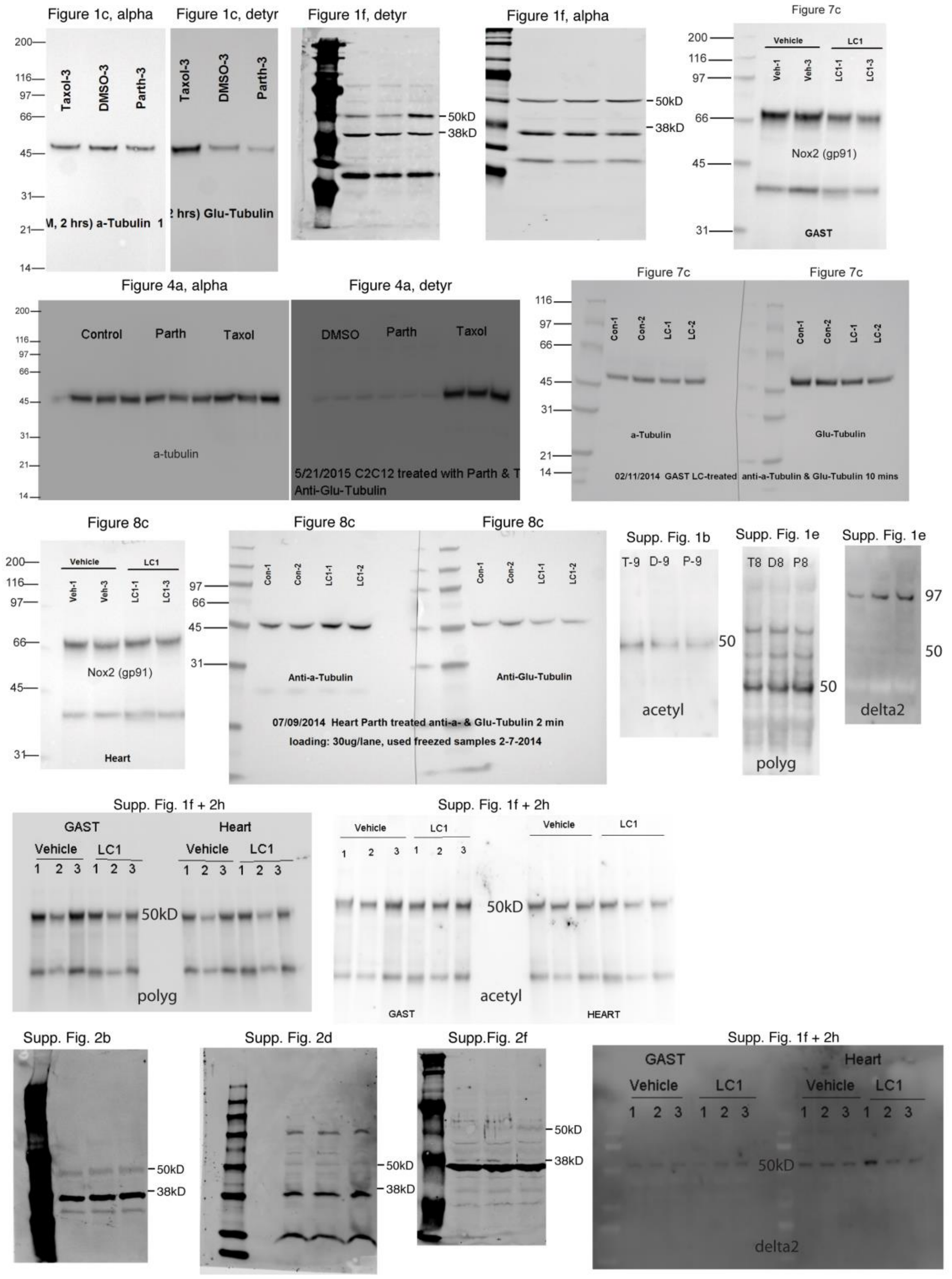

Supplementary Figure 6: Western blots used in Main and Supplemental Figures.
